# Natural Compound Modulates the Cervical Cancer Microenvironment—A Pharmacophore Guided Molecular Modelling Approaches

**DOI:** 10.3390/jcm7120551

**Published:** 2018-12-15

**Authors:** Shailima Rampogu, Doneti Ravinder, Smita C. Pawar, Keun Woo Lee

**Affiliations:** 1 Division of Life Science, Division of Applied Life Science (BK21 Plus), Plant Molecular Biology and Biotechnology Research Center (PMBBRC), Research Institute of Natural Science (RINS), Gyeongsang National University (GNU), 501 Jinju-daero, Jinju 52828, Korea; shailima.rampogu@gmail.com; 2 Department of Genetics, University College of Science, Osmania University, Hyderabad 500 007, Telangana, India; ravi4bios@gmail.com

**Keywords:** natural compounds, aromatase inhibitors, forskolin, Bcl-2, Bax, Caspase3, pro-inflammatory genes, pharmacophore modelling

## Abstract

Cervical cancer is regarded as one of the major burdens noticed in women next to breast cancer. Although, human papilloma viruses (HPVs) are regarded as the principal causative agents, they require certain other factors such as oestrogen hormone to induce cervical cancer. Aromatase is an enzyme that converts androgens into oestrogens and hindering this enzyme could subsequently hamper the formation of oestrogen thereby alleviating the disease. Accordingly, in the current investigation, a structure based pharmacophore was generated considering two proteins bearing the Protein Data Bank (PDB) codes 3EQM (pharm 1) and 3S7S (pharm 2), respectively. The two models were employed as the 3D query to screen the in-house built natural compounds database. The obtained 51 compounds were escalated to molecular docking studies to decipher on the binding affinities and to predict the quintessential binding modes which were affirmed by molecular dynamics (MD) simulations. The compound has induced dose-dependent down regulation of PP2B, Nitric oxide synthase-2 (NOS2), and Interleukin 6 (IL-6) genes in the HeLa cells and has modulated the expression of apoptotic genes such as Bax, Bcl2, and caspases-3 at different concentrations. These results guide us to comprehend that the identified aromatase inhibitor was effective against the cervical cancer cells and additionally could server as scaffolds in designing new drugs.

## 1. Introduction

Cervical cancer is regarded as one of the major burdens noticed in women next to breast cancer claiming nearly about 250,000 lives annually and is reported to increase exponentially [1]. Notably, human papilloma viruses (HPVs) are regarded as the major etiological factors for initiating the disease besides being associated with vaginal cancers, head and neck cancer [2,3,4]. The characteristic link between the HPVs and the cervical cancer was postulated by Harold zur Hausen in the early 1970’s [5,6]. Structurally, the HPVs contain the circular non-enveloped DNA of approximately 8 kb which can be divided into three segments. The regulatory region is critical in deducing the host range and the tissue tropism of each virus type. Additionally, this region possesses the origin of viral replication and several cellular transcriptional factor binding sites thereby governing the viral gene expression post infection [6]. Furthermore, the genome consists of early region and the late region that encodes E1, E2, E4, E5, E6, and E7 proteins and L1 and L2 proteins, correspondingly. The proteins E6, E7, and E5 are the oncoproteins executing the cell transformation events [6,7]. Interestingly, HPV alone cannot induce cervical cancer implying the necessity of other factors such as hormones to provoke cervical cancer [8,9]. 

Oestrogen is the major female reproductive hormone responsible for performing several biological and physiological functions in women [10] and the uterine cervix is highly responsive to the oestrogen. Moreover, the high degree of cervical cancer cases are reported in women who are in their premenopausal age and the women who are on oral contraceptives [2,11,12]. The association of oestrogen is implicated with a host of diseases including cancers predominantly breast cancer. Nevertheless, the oestrogen and the oestrogen α in the cervix modulate the cervical epithelial cell proliferation and differentiation in response to high levels of the hormone [4] inferring that the uterine cervix is responsive to oestrogen [4]. Several reports evidenced the role of oestrogen in the development of cervical cancer [2,4,13,14,15] redirecting the need for the development of antiestrogen drugs.

Although, treatments such as surgery, radiotherapy and chemotherapy have been traditionally used, their efficacy has been compromised in the advanced and recurrent cases. Under such conditions, use of aromatase inhibitors offers a promising alternative that specifically diminishes the elevated levels of oestrogen. Aromatase is an enzyme that converts the androgens to oestrogens and is principally located in the ovaries [16,17]. Furthermore, the overexpression of aromatase is evinced in certain diseases [18,19,20] and is chiefly attributed with breast cancer. Moreover, it is well documented that in the cervical cancer the oestrogen levels and the aromatase levels are elevated [8], subsequently conferring it as a valuable target to design novel drugs. 

The use of natural compounds as anticancer drugs is gaining wider attention due to their abundance in availability and reduced side effects [21,22,23,24]. The herbal extracts were typically able to act against viral oncogenes and hinder the cooperative signaling and deregulation of host cell gene expression [25]. Tumour cells are targeted by natural compounds by governing apoptosis pathways and autophagic pathways, suppressing the NF-κB and angiogenesis inhibition in prostate cancer [26,27]. Moreover, a variety of natural compounds were targeted against the HPV E6 oncoprotein [23,28]. 

Delineating the microenvironment of cancer, it can be understood that, rapid cancer growth leads to tumor hypoxia and nutrient deprivation, which promotes chronic inflammatory feed-forward signalling and selection of resistant tumors that are clinically challenging and sometimes untreatable. Several pro-inflammatory proteins such as COX2, NF-κB, IL-6, IL-8, S100 calcium binding protein A8 (S100A8), and vascular endothelial growth factor (VEGF) are markers of chronic inflammation in the tumour microenvironment. IL-6 is a multifunctional cytokine that was originally characterized as a regulator of immune and inflammatory responses; however, elevated expression of IL-6 has been detected in multiple epithelial tumors. Correspondingly, IL-6 is regarded as an important tumour promoting factor in various types of human cancer including glioma, lymphoma, melanoma, as well as breast, ovarian, pancreatic, prostate, renal, and colorectal cancer. Apoptosis or programmed cell death is critically involved in normal cell turnover, hormone-dependent atrophy, immune system and chemical-induced cell death, etc. This mechanism is controlled by several molecules regulated intrinsically or extrinsically through death signals from the outside of the cell. Notably, the gene families such as, Bcl-2, Bax and caspases are widely known to trigger apoptosis. Calcineurin is a calcium/calmodulin-dependent serine/threonine phosphatase that has been involved in many cellular signalling processes such as synaptic plasticity and memory formation, T-cell activation and apoptosis, muscle growth and differentiation, and cardiac functions. Calcineurin plays a role in apoptosis via inducing the cytochrome c/caspases dependent pathway and by dephosphorylating Bad, a pro-apoptotic member of Bcl-2 family. Recent studies have also demonstrated the involvement of calcineurin in the cell cycle by regulating the cdk4 (cyclin dependent kinase4), a G0/G1 checkpoint element. Inflammation is a major component of the tumour microenvironment and a driving force in cancer initiation, promotion, and progression.

Correspondingly, motivated by these reports, in the current investigation we have attempted to screen the ligands from the natural compounds against aromatase that confer with the potential inhibitory features employing the structure-based pharmacophore approach. Subsequently, the identified compound has been assessed in vitro to probe into their efficacy against cervical cancer HeLa cells. 

## 2. Methods

### 2.1. Structure Based Pharmacophore Generation

Structure based pharmacophore (SBP) is regarded as one of the superlative methods adapted in the field of drug discovery that exploits the features of protein and its corresponding ligand interactions thereby imparting knowledge on the key essential features required for inhibition. For the current study, SBP was generated considering two proteins, the aromatase protein bearing the PDB code 3EQM with the natural substrate 4-androstene-3-17-dione as cocrystal and the protein with the PDB code 3S7S in complex with the drug exemestane. In order to generate the pharmacophore models, the residues in vicinity to the ligand have been considered.

Accordingly, the *clean protein* protocol was enabled to check for any gaps existing in the protein. Subsequently, the *receptor ligand pharmacophore generation* module embedded with the Discovery Studio v.18 (DS) was chosen. The parameter for maximum pharmacophore was chosen as 10 with minimum and maximum features as 4 and 6 respectively. Correspondingly, the maximum charge distance was opted as 5.6 Å with an interfeature distance of 2.0 Å, while retaining the maximum hydrogen bond distance and maximum hydrophobic distance as default. The maximum hydrogen bond distance was set as 3.0 Å.

### 2.2. Validation of the Generated Pharmacophore Model

Model validation is one of the essential steps to assess the robustness of the pharmacophore models in redeeming the active compounds from the inactive compounds. The decoy set method of validation was conducted by instituting an external dataset (D) comprising of 1500 with 20 active compounds. Subsequently, the ligand pharmacophore mapping module furnished with the DS was enabled with *rigid* fitting method. The obtained results were assessed based upon the enrichment factor (EF) and goodness of fit (GF) scores employing the formula:

(1) $$\mathrm{EF}=\frac{\mathrm{Ha}\times D}{\mathrm{Ht}\times A}$$

(2) $$\mathrm{GF}=\left(\frac{\mathrm{Ha}}{4\mathrm{HtA}}\right)\left(3A+\mathrm{Ht}\right)\times \left\{1-\frac{\mathrm{Ht}-\mathrm{Ha}}{D-A}\right\}.$$

### 2.3. Virtual Screening of Natural Compounds Database Using Two Pharmacophore Models

The validated pharmacophore models have been used has the 3D query to retrieve the compounds imbibed with the key inhibitory chemical features. Accordingly, a database consisting of 1330 compounds procured from different literatures and Naturally Occurring Plant-based Anti-cancer Compound-Activity-Target (NPACT) (http://crdd.osdd.net/raghava/npact/) database that is uniquely composed of naturally occurring plant based anticancer compounds was formulated. Logically it is assumed that small molecules that represent the features of the pharmcophore might be imbibed with the key inhibitory features. Accordingly, the ligand pharmacophore mapping module embedded with the DS was employed with rigid fitting method.

### 2.4. Molecular Docking Studies

Molecular docking is one of the important criteria in the field of structural molecular biology and computational drug designing that predominantly predicts the binding mode of the ligands and ranks them based upon the scores [29]. For the current investigation, the CDOCKER (CHARMm-based DOCKER) equipped with the DS was employed that operates on the CDOCKER algorithm utilizing the CHARMm [30]. This is a grid based molecular docking protocol wherein the receptor is held firm allowing the ligands to wobble during the refinement. Mechanistically, ligand conformations were arbitrarily generated applying the high temperature molecular dynamics and were then randomly rotated. These conformations were refined by grid-based simulated annealing. The ligands were ranked and are further sorted in accordance with the -CDOCKER interaction energy. Logically, higher the -CDOCKER interaction energy greater is the binding affinity between the protein ligand complex. The target protein for the current study is aromatase with the PDB code 3EQM which is in complex with androstenedione. The binding site is defined for all the atoms at 10 Å around the cocrystal. Accordingly, the residues Arg115, Ala306, Asp309, Val370, Leu372, Met374, and Leu477 were labelled as critical residues for exerting inhibitory activities. Each ligand was allowed to generate 50 conformations. The best pose was determined based upon the ideal binding mode, highest dock score from the largest cluster and key residue interactions between proteins. The selected compounds were upgraded to molecular dynamics (MD) simulations to probe into the stability of the protein and to decipher on the molecular interactions.

### 2.5. Molecular Dynamics Simulations to Elucidate the Binding Mode of the Compounds

Molecular dynamics (MD) studies have been extensively employed to delineate on the molecular motion, predict the enzyme mechanism and further to comprehend on the complex assemblies. The MD simulations additionally disseminates knowledge on the nature of the small molecules with its protein at the atomistic level [31,32]. For the current investigation, MD was employed to assess the stability of the target and the ligand complexes and were analysed according to the root mean square deviation (RMSD), radius of gyration (Rg) and the potential energies recruiting GROningen MAchine for Chemical Simulations (GROMACS v5.0.6, www.gromacs.org). The MD was commenced considering the ideal binding modes of the aromatase in complex corresponding inhibitors from the molecular docking results utilizing all atom CHARMm27 force field [33] and retrieving the ligand topologies from SwissParam [34]. A dodecahedron water box [35] was generated and was solvated with TIP3P water model and were neutralized with counter ions. The steepest descent energy minimization algorithm was used to relax the initial structures by evading the rigid hindrances. Additionally, the number of steps was confined to 10,000 using the minimization force less than 10,000 kJ/mol. Thereafter, a twofold equilibration was applied by NVT and NPT, respectively. The first step of equilibration was conducted with a constant number of particles, volume, and temperature complex (NVT) at 300 K for 1 ns monitored by V-rescale thermostat. The second equilibration step was performed for number of particles, pressure, and temperature (NPT) ensemble for 1 ns controlling the pressure at 1 bar with Parrinello–Rahman barostat [36]. During the equilibration steps the backbone of the protein was refrained, while permitting the movement of the solvent molecules and the counter ions. The equilibrated ensembles were subjected to MD simulations conducted for 50 ns employing LINCS and SETTLE [37,38] algorithm for bond constraints and geometry of water molecules. Particle Mesh Ewald (PME) [39] method was used to calculate the long-range electrostatic interactions by defining a cut-off value of 9 Å and 14 Å for short-range interactions and van der Waals interactions, correspondingly. The obtained results were evaluated employing visual molecular dynamics (VMD) [40], GROMACS, and DS. The identified compound is labelled as Forskolin (FSK).

### 2.6. In Vitro Assay for Evaluating the Effect of the Drug on Cancer Physiology/Micro Environment

In order to assess the impact of the identified drug on the HeLa cells, we targeted specific genes that are known to assist the cervical cancer progression. Particularly, we focused on modulation of PP2B, IL-6 and NOS2 proteins and some of the apoptotic proteins Bax, Bcl2 and Caspase-3.

### 2.7. Procurement of the Material

HeLa cells were commercially sourced from NCCS (National Centre for Cell Science, Pune, India) in mycoplasma free condition. DMEM with high glucose, foetal bovine serum (FBS) and PBS from Gibco BRL (Carlsbad, CA, USA). The chemicals and reagents were purchased from Merck, nitro cellulose membrane was obtained from Millipore (Bangalore, India). The antibodies Bax, Bcl2, Caspase-3 were sourced from Cell Signalling Technologies, PP2B, IL-6 and NOS2 primary antibodies are purchased from Santa Cruz biotechnologies (Dallas, TX, USA). Correspondingly, Forskolin purchased from sigma (St. Louis, MO, USA) and the 10 mM stock of Forskolin (M.W. 410.5) was prepared with DMSO and all sub stocks and working stocks were prepared with 1 × PBS. 

### 2.8. Maintenance of Mammalian Cell Culture

The procured HeLa cells were maintained in Dulbecco’s modified Eagle’s medium (DMEM), supplemented with 10% heat inactivated FBS and antibiotics, 100 IU/mL penicillin, 100 µg/mL streptomycin and 2 mM L-glutamine. The cells were maintained at 37 °C with 5% CO_2_ and 95% relative humidity and sub cultured approximately once in every three days. 

### 2.9. Cytotoxicity Assay

In-vitro cytotoxic activity against different dose dependent treatments of Forskolin in HeLa cancer cell line was performed using 96-well culture plates in triplicates. To each well the 8 × 10^3^ cells/well were seeded and were allowed to grow at 37 °C for overnight in 5% carbon dioxide incubator (Thermo Fisher Scientific, Waltham, MA, USA). On the following day, a different concentration (doses i.e., FSK-10μM, FSK-20μM, FSK-40 μM, FSK-80 μM and FSK-100 μM) of FSK was added. The plates were further incubated for 24 h and 100 µL of 10% (TCA) trichloroacetic acid was added gently to cease the cell growth by thin layering of trichloroacetic acid into each well. The plates were further incubated at 37 °C for 1 h to fix the cells attached to the bottom of the wells. The plates were washed five times with distilled water to remove traces of medium, trichloroacetic acid, sample, serum proteins, and then air dried. The cell growth in air dried plates was measured by staining with 100 µL (0.057% *w*/*v* in 1% *v*/*v* Acetic acid) of sulforhodamine B dye for 30 mins and then rinse the plate for four times in 1% *v*/*v* acetic acid to remove unbound dye. Then add 100 µL/ well of Tris-base buffer (0.01 M, pH 10.4) and plates were stirred for 5 min on a mechanical shaker. The optical density was measured at 540 nm on ELISA plate reader (Bio-Rad Laboratories, Hercules, CA, USA)

### 2.10. Cell Cycle Analysis

HeLa cells (10 × 10^5^) were seeded in a 60 mm Petri dish and were allowed to grow for 24 h. FSK of FSK-20 μM, FSK-40 μM, FSK-80 μM and FSK-100 μM concentrations were added to the culture medium and the cells were incubated for an additional 24 h. Thereafter, the cells were harvested with trypsin/ Ethylenediaminetetraacetic acid (EDTA), fixed with ice-cold 70% ethyl alcohol (EtOH) at −80 °C for overnight. On the next day the fixed cells were washed with PBS and incubated with 1 mg/mL RNase A solution (Sigma) at 37 °C for 30 min. Subsequently, the cells were collected by centrifugation at 2000 rpm (Heraeus Sorvall swinging bucket rotor (model #75002000), max speed: 4700 rpm, Heraeus Multifuge 1S-R, Thermo Scientific) for 5 min and further stained with 250 µL DNA staining solution (10 mg propidium iodide (PI), 0.1 mg trisodium citrate, and 0.03 mL Triton X-100 dissolved in 100 mL sterile Milli-Q water at room temperature for 30 min in the dark). The DNA content of 20,000 events was measured by flow cytometry (DAKOCYTOMATION, Beckman Coulter, CA, USA). The resultant histograms were analysed using Summit software.

### 2.11. Western Blot Analysis

HeLa cells (5 × 10^5^) were seeded in a 60 mm Petri dish and were allowed to grow for 24 h. FSK of FSK-2 μM, FSK-5 μM, and FSK-10 μM concentrations were added to the culture medium and the cells were incubated for an additional 24 h. Thereafter, the cells were harvested with trypsin/EDTA, and then western blot analysis was executed based upon Sambrook et al. (1989) [41]. To prepare the whole cell extract, cells were washed with PBS and suspended in lysis buffer (20 mM Tris, 1 mM EDTA, 150 mM NaCl, 1% NP 40, 0.5% deoxycholic acid, 1 mM β-glycerophosphate, 1 mM sodium orthovanadate, 1 mM phenylmethylsulfonyl fluoride (PMSF), 10 μg/mL leupeptin, 20 μg/mL aprotinin). After 30 min of shaking at 4 °C, the mixtures were centrifuged (14,000 rpm) for 40 mins, and the supernatants were collected as the whole-cell extracts. The protein content was determined according to the Bradford method. An equal amount of total cell lysate was resolved on 10 % sodium dodecyl sulfate- Polyacrylamide gel electrophoresis (SDS PAGE) gels along with protein molecular weight standards, and then transferred onto nitrocellulose membranes. The membranes were blocked with 5% *w*/*v* non-fat dry milk and then incubated with various primary antibodies of PP2B (Calcineurin), NOS2, IL-6, Bcl2, Bax, and Caspase-3 in 10 mL of antibody-diluted buffer (1 × Tris-buffered Saline and 0.05% Tween 20 with 5% milk) with gentle shaking at 4 °C for 8–12 h and then incubated with peroxidase conjugated secondary antibodies. The signals were detected using an ECL Western blotting detection kit (Bio-Rad Laboratories, Hercules, CA, USA)

### 2.12. Statistical Analysis

All experiments including immunoblots were performed in triplicates. The mean and standard deviation were calculated from the triplicates. For immunoblot analysis, the mean intensity of the bands was measured using ImageJ software (NIH, Bethesda, MD, USA.). Using mean and standard error (SEM), *p*-values were calculated by one-way ANOVA using Sigmaplot 12.3 software (San Jose, CA, USA)

## 3. Results

### 3.1. Structure-Based Pharmacophore Generation

**Figure 1:**
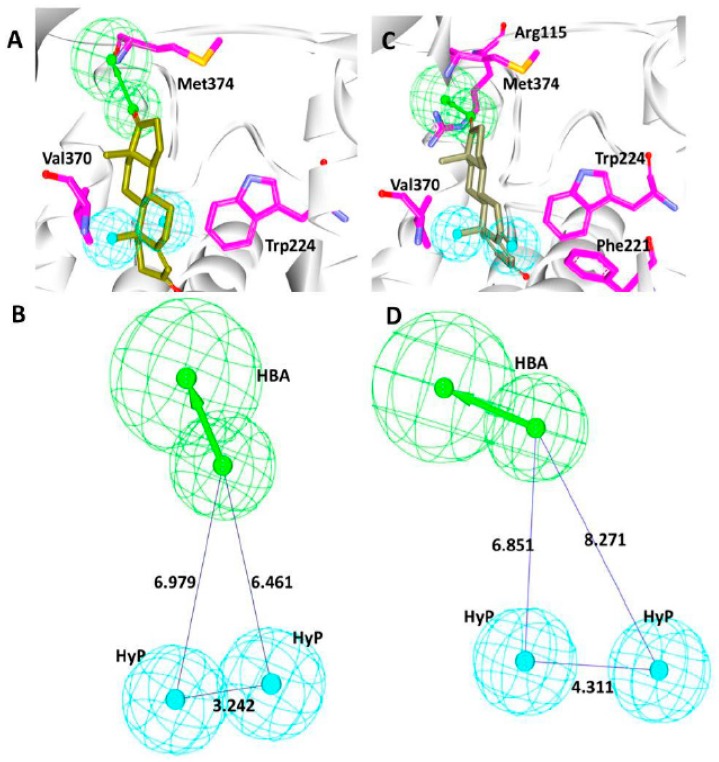
Pharmacophore generation. (**A**) pharm 1 with key residues complementary to the target 3EQM, (**B**) pharm 1 with interfeature distance, (**C**) pharm 2 with key residues complementary to the target 3S7S, and (**D**) pharm 2 with interfeature distance.

The structure-based pharmacophore precisely foretells the inhibitory key features complementary to the residues. Accordingly, the target structures for the current study to generate the pharmacophore are 3EQM and 3S7S. The protein 3EQM (pharm 1) has an innate natural substrate androstenedione while the structure 3S7S (pharm 2) is in complex with the approved drug exemestane. Structurally, the drug exemestane is an analogue of the substrate and occupies the binding site in a similar fashion as that of the substrate, implying that both the structures may generate a similar kind of pharmacophore model. Accordingly, both the targets have generated three featured pharmacophore model consisting of one hydrogen bond acceptor and two hydrophobic features. The key residue Met374 has represented hydrogen bond acceptor feature in pharm 1, Figure 1A, while in pharm 2 it was complimentary to the key residue Met374 and Agr115, Figure 1C. Furthermore, it was interesting to note that the one of the hydrophobic features in pharm 1 and pharm 2 was displayed for the key residue Val370, while the other hydrophobic feature was complementary to Trp224 in pharm 1 and Phe221 in pharm 2, respectively, Figure 1A,C, with a marginal variation in their interfeature distance as depicted in Figure 1B,D.

### 3.2. Pharmacophore Validation

Decoy set method of validation was fundamentally executed to determine the ability of the pharmacophore in retrieving the active compounds from a given database. The pharm 1 and pharm 2 were upgraded to retrieve the active compounds from the dataset initiating the *ligand pharmacophore mapping* tool embedded with the discovery studio v.18 (DS). Correspondingly, pharm 1 has retrieved 14 compounds (Ht) consisting of 13 actives (Ha). Consequently, the goodness of fit (GF) value is computed as 0.73 and EF value of 69.64. Pharm 2 has redeemed 16 compounds (Ht) with 13 actives (Ha) generating a GF score of 0.75 and EF score of 60.93. The GF score serves as the decisive factor in evaluating the quality of the pharmacophore and ranges between 0 to 1 representing the null and ideal model. The current pharmacophore models have generated a GF score of 0.73 and 0.75 which is in proximity to the ideal model value 1. Therefore, these models can be considered as a good model for further studies, Table 1.

### 3.3. Retrieving the Compounds with the Pharmacophore Features

In order to retrieve the prospective candidate compounds imbibed with inhibitory chemical features, the pharmacophore models, pharm 1 and pharm 2 were used as the 3D query to screen the natural compounds database consisting of 1330 compounds enabling the ligand pharmacophore mapping module accessible on the DS. Correspondingly, pharm 1 has retrieved 1172 compounds and pharm 2 has redeemed 1165 compounds which were further filtered by setting the fit value at 2.7. Consequently, the obtained 618 compounds from pharm 1 and 575 compounds from pharm 2 were evaluated for the presence of the common compound. This search has yielded 51 compounds, rationally implying that these compounds could pose as the better alternative therapeutics. Therefore, these compounds were upgraded to the molecular docking studies and molecular dynamics simulations to assess their behaviour at the atomic level and interpret the molecular mechanism of inhibition.

### 3.4. Screening by Molecular Docking Studies

Molecular docking is one of the remarkable methods employed to sample the conformations of small molecules at the proteins active site adapting various scoring functions. The procured 51 compounds were escalated to molecular docking studies to assess the binding affinities between the protein and the ligand and additionally to gauge on the quintessential binding mode. To secure the most potential prospective drug, the compound exemestane has been considered as the reference compound. Furthermore, all the compounds that have displayed higher dock score than the reference compounds were upgraded for further assessment. Consequently, the compounds from the largest cluster were critically probed for the key residue interactions demonstrating an ideal binding mode. A total of nine compounds have obeyed to the above criteria and were therefore labelled as potential hits, Supplementary Table S1. Amongst them, the top ranked compound (FSK) was commercially sourced and tested in vitro against the cervical cancer HeLa cell lines to test its efficacy.

### 3.5. Elucidation of the Binding Mode by Molecular Dynamics Studies

To additionally gain insight into the nature of the small molecules at the proteins active site and further to evaluate the stability of the systems correspondingly affirming the molecular docking results, the molecular dynamics simulation was executed. In order to accomplish this endeavour, GROMACS package was adapted for 50 ns run and the obtained findings were analysed based upon the RMSD for the protein backbone atoms, protein ligand complex, Rg and potential energy profiles. Accordingly, the compound protein-FSK complex was thoroughly evaluated by molecular dynamics studies. The RMSD profiles have unravelled that the system was relatively stable below 0.2 nm with an average being 0.17 nm. Upon comparing the stability profiles with the protein ligand complex it was observed that the plots were stable below 0.25, projecting an average of 0.22 nm, Supplementary Figure S1A. These results have disclosed that the system displayed a phenomenal stability and the same was affirmed by the potential energy profiles, Supplementary Figure S1B and Rg Supplementary Figure S1C. 

**Figure 2:**
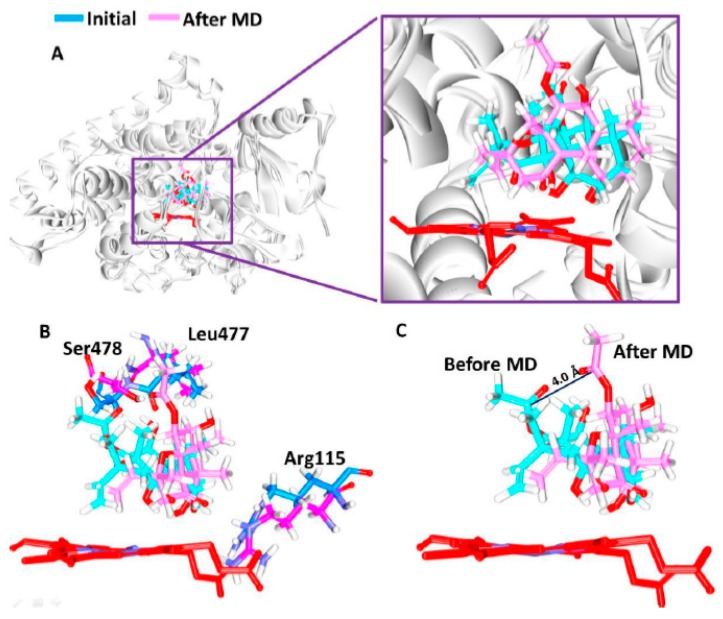
Accommodation of the ligand at the active site of the protein. (**A**) The ligand position before and after the molecular dynamics (MD) simulations. The ligand occupies in the substrate binding site. (**B**) The close residues appear to be unchanged. (**C**) The ligand moved 4 Å during the simulation.

To determine the reliable binding mode of the protein with the compound, the trajectories from the last 3 ns were obtained. Upon subsequent superimposition, it was disclosed that the compound has occupied the same binding site of the substrate portraying a similar binding fashion, Figure 2A and supplementary Figure S2. Notably, no striking change in the positioning of the interacting residues was recorded before and after the MD simulations, Figure 2B and Table 2.

**Figure 3:**
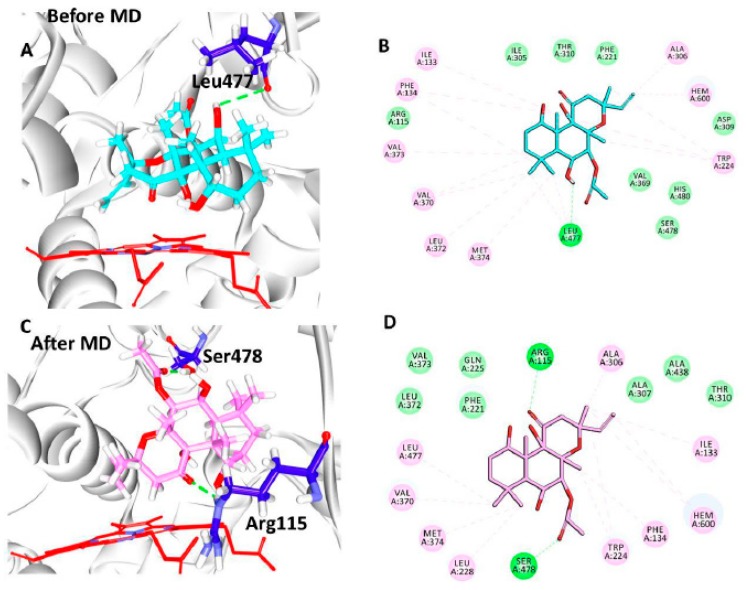
Intermolecular interactions between the protein and the ligand. (**A**) Only one hydrogen bond during the docking simulations. (**B**) Comprehensive intermolecular interactions. (**C**) After the MD simulations for 50 ns the ligand has formed two hydrogen bonds. (**D**) Comprehensive intermolecular interactions.

Delineating on the intermolecular interactions, it was observed that the best molecular dock pose has generated one hydrogen bond with the key residue Leu477 rendered by an acceptable bond length of 2.9 Å, Figure 3A,B. The ligand was held by Arg115, Phe221, Ile305, Asp309, Thr310, Val369, His480, and Ser478 contributed by the van der Waals interactions and the residues Ile133, Phe134, Trp224, Ala306, Val370, Leu372, Val373, and Met374 demonstrated the π-π/π-alkyl interactions, Figure 3A,B.

Focussing on the ligand position post MD, it was observed that the ligand has displayed approximately 4 Å from its initial position thereby forming two hydrogen bonds conferred by the residues Arg115 and Ser478 demonstrating an acceptable bond length of 2.0 Å and 1.9 Å, Figure 2C respectively, and was consistent at the position. Correspondingly, the ligand was held firm at the position with the residues Phe221, Gln225, Ala307, Thr310, Leu372, Val373, and Ala438 through the van der Waals interactions. Furthermore, the residues Ile133, Phe134, Tpr224, Leu228, Ala306, Val370, Met374, and Leu477 have aided in positioning the ligand at the active site of the protein via π-π/π-alkyl interactions, Table 2, Figure 3C and 3D. The pre MD hydrogen bond interaction demonstrated by the Leu477 was missing and the residue has formed the hydrophobic alkyl interactions. The reference compounds has formed two hydrogen bonds, the HH11 atom of Arg115 and HN atom of Met374 have interacted with the O1 atom of the ligand rendered by a bond length of 2.5 Å and 1.9 Å, respectively. Additionally, the residues Phe134, Ile305, Ala306, Asp309, Thr310, Leu477, Ser478 and Ile133, Phe221, Val370, Val373 have locked compound by van der Waals interactions and π-π/π-alkyl interactions, Table 2. Contemplating on the intermolecular interactions, it can be noted that the FSK has made a stable inter action with the Arg115 as was seen with the reference and additionally, Ile133 was also observed forming the π/π-alkyl interaction. This finding lead us to comprehend that the movement of the ligand approximately by 4 Å was significant in attaining the stable position at the active site of the protein and further gaining the hydrogen bond with the key residue Arg115. However the residues involved in the hydrogen bond have shown no variation before and after the MD, Figure 2B. Deciphering on the hydrogen bonds interactions during the MD simulations, it was revealed that the protein-ligand has formed the stable hydrogen bond interaction with the average being 1.07, Supplementary Figure S1D. Comprehending on the key residues interactions in the post MD results, it was vivid that they have participated in holding the ligand at that the active sites either by van der Waals interactions or via π-π/π-alkyl interactions. The residue Ala306 has interacted with the C25 atom of the ligand represented by an alkyl hydrophobic bond rendered by a distance of 3.9 Å. Another key residue Val370 has prompted an alkyl hydrophobic bond with a distance of 4.5 Å. The residue Met374 interacted to the C22 atom of the ligand through an alkyl hydrophobic bond by a distance of 3.6 Å. The residue Leu477 also conferred with an alkyl hydrophobic bond rendered by a distance of 4.3 Å. Furthermore, the key residue Leu372 has generated van der Waals interaction. These results guide us to deduce that the key residues have involved with the ligand interaction thereby illuminating its suitability as a potential cervical cancer inhibitor.

Furthermore, the retrieved compound was identified as Forskolin (FSK) upon searching the databases and has imbibed with the two pharmacophore features, Supplementary Figure S3.

### 3.6. Maintenance of Mammalian Cell Culture

**Figure 4:**
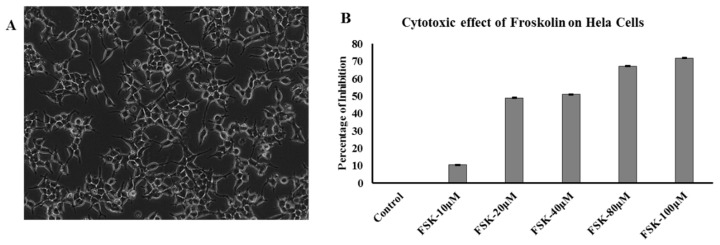
HeLa Cells and Cytotoxicity Assay. (**A**) HeLa Cell Morphology were seen by confocal Microscopy. (**B**) Hela Cells were treated with Forskolin with different concentrations FSK-10 µM, FSK-20 µM, FSK-40 µM, FSK-80 µM, and FSK-100 µM for 24 h. The SRB cell cytotoxic assay was conducted and optical density (OD) was observed at 540 nm. Control indicates untreated cells, and assumes as 0% inhibition. (Error bars represents the standard deviation form triplicates of each analysis).

The parental cell line HeLa was maintained in healthy and mycoplasma-free status successfully. During the drug treatment, an untreated cell in one flask was maintained as running culture along with the treated flasks, Figure 4A.

### 3.7. Effect of FSK on Hela Cells (Cytotoxicity Assay)

These FSK-10 μM, FSK-20 μM, FSK-40 μM, FSK-80 μM, and FSK-100 μM doses were evaluated for their cytotoxicity in human cervical cancer cell line (HeLa) by using the sulforhodamine B (SRB) method. These doses exhibit IC_50_ at FSK-20 μM are considered to be active on the cervical cancer HeLa cells. On other hand, FSK-40 μM, FSK-80 μM, and FSK-100 μM doses were showing dose dependent cyto-toxicity on HeLa cells. Froskolin was showing cytotoxicity activity at lower concentrations only. Dose of FSK-20 μM exhibited significant cytotoxicity activity, Figure 4B. 

### 3.8. Cell Cycle Analysis

**Figure 5:**
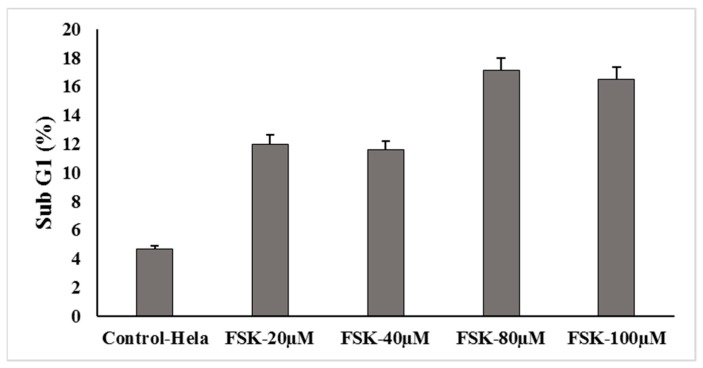
Cell cycle analysis. The graph depicting the percentage of apoptosis with treatment of Forskolin.

**Figure 6:**
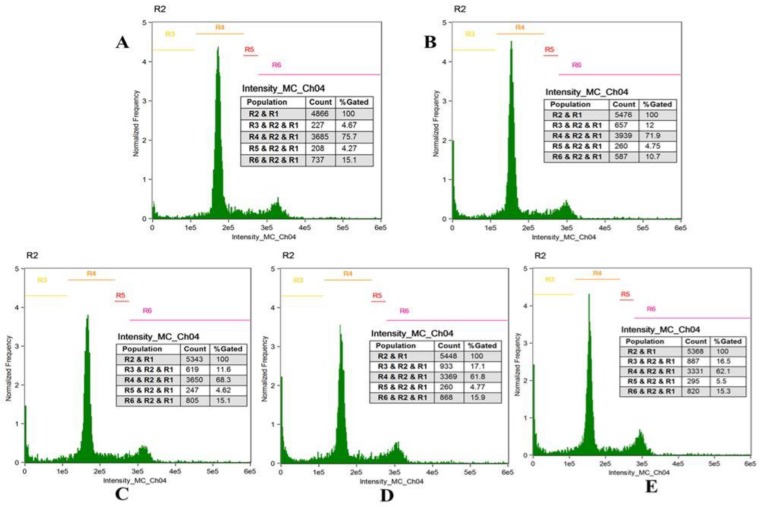
Fluorescence-activated cell sorting analysis of the cell cycle in Forskolin-treated HeLa with respect to untreated cells. (**A**) Hela untreated cells, (**B**) Forskolin-20 µM, (**C**) Forskolin-40 µM, (**D**). Forskolin-80 µM, (**E**) Forskolin-100 µM.

In order to understand the effect of Forskolin on cell cycle in Hela cells, Fluorescence-activated cell sorting (FACS) analysis was conducted for some representative concentrations of forskolin in Hela cervical cancer cells. The cells were treated for 24 h with FSK-20 μM, FSK-40 μM, FSK-80 μM and FSK-100 μM concentrations. It was observed that cells showed no significant cell cycle arrest (i.e., cells accumulated in G1phase), even upon increasing the concentration of the FSK compound; however the percentage of apoptotic cells was enhanced, Figure 5 and Figure 6.

### 3.9. Western Blot Analysis of PP2B, IL-6 and NOS2 Genes

**Figure 7:**
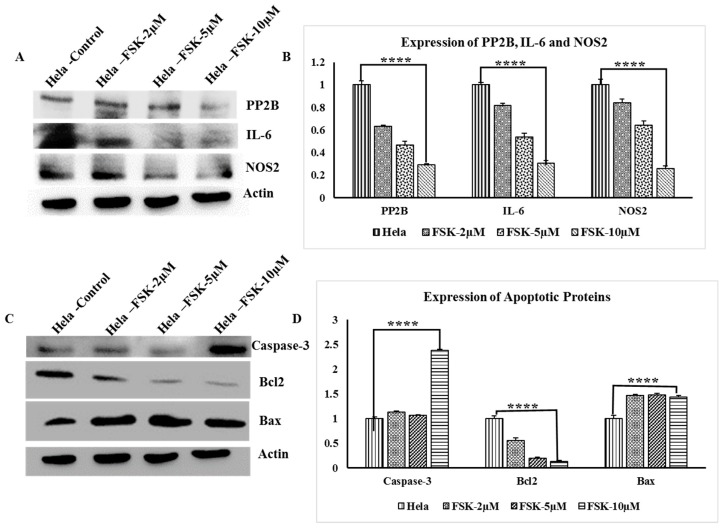
Western blot results: (**A**). Expression Analysis of PP2B, Interleukin-6 and iNos (NOS2) upon treatment of Forskolin Compound. Treatments in HeLa (2 µM, 5 µM, and 10 µM) Cell lysates were subjected to Western blot analysis with PP2B, IL-6, and NOS2 antibody followed by sequential re probing against Actin. (**B**) Bar graph below indicates densitometric analysis of bands in immunoblot. (**C**) Expression Analysis of Caspase-3, Bcl2 and Bax upon treatment of Forskolin Compound HeLa and Forskolin-Treatments in HeLa (2 µM, 5 µM, and 10 µM) Cell lysates were subjected to Western blot analysis with Caspase-3, Bcl2 and Bax antibody followed by sequential re probing against Actin. (**D**) Densitometric analysis of the bands in Western blot, (**** denotes *p* < 0.0001).

Expression of PP2B, IL-6 and NOS2 in HeLa cells upon Forskolin Treatments with FSK-2 μM, FSK-5 μM, and FSK-10 μM. Western blot analysis showed that PP2B, IL-6 and NOS2 were down-regulated in dose dependent manner; Actin was reported as internal control, Figure 7A.

### 3.10. Analysis Apoptotic Proteins

Apoptosis protein analysis of Bcl-2, Bax, and Caspase-3 protein was determined by Western blot analysis after overnight incubation with treatment of FSK-2 μM, FSK-5 μM, and FSK-10 μM of Forskolin on HeLa cervical cancer cells. Actin was reported as internal control. It is evident that the incubation with Forskolin caused relative increase in caspase-3 at FSK-10 μM concentration. Likewise, Bax also has demonstrated a relative increase in its expression in a dose dependent as Bax is pro-apoptotic protein from Bcl-2 family. The Bcl-2 protein expression was dose-dependently down-regulated with Forskolin treatment in HeLa cancer cells, as this Bcl2 is an anti-apoptotic protein from Bcl-2 family, Figure 7B.

## 4. Discussion

It is well documented that over expression of oestrogen leads to several pathological diseases including cervical cancer in women [4]. Predominantly, the oestrogen is generated due to the enzymatic action of aromatase on androgens. Furthermore, it is reported that oestrogen plays a pivotal role in HPV persistence and subsequent neoplastic progression by elevating the viral gene expression [4]. Logically, inhibiting the formation of oestrogen leads to diminished levels of oestrogen thereby mitigating the disease. Accordingly, we attempted to identify a natural compound that represents the pharmacophore features of the aromatase substrate and the drug. Consequently, the Forskolin was redeemed representing the inhibitory features of the cocrystal. 

The MD studies have unravelled that the compounds occupies the active site and forms a stable bond with the key residue Agr115 along with Ser478. It is worth discussing that the FSK has occupied the active site throughout the simulations and has moved to its stable state after 15 ns by 4 Å. This resulted in the formation a hydrogen bond with the important residue Arg115. It was earlier established that the involvement of the residues Agr115 and Ser478 were noticed with the natural flavonoid compounds as aromatase inhibitors [42] thereby illuminates the potentially of FSK [43]. 

Further, the therapeutic ability of FSK was assessed in vitro, to authenticate its efficiency. Correspondingly, HeLa cells were treated with FSK and probed for its effect on potent established cancer markers like PP2B, IL-6, NOS2, Caspase3, Bcl2, and Bax. 

The calcineurin, serine-threonine phosphatase (PP2B), is increasingly being recognized as a commonly activated target in cancer. Activation of calcineurin is implicated in signalling pathways promoting proliferation, migration, and metastasis [44,45] and is involved in apoptosis [46]. Therefore, effect of FSK on PP2B was assessed and upon treatment resulted in downregulation of PP2B in a dose dependent manner. Interleukin-6 (IL-6) a proinflammatory cytokine known to be associated with cervical cancer and acts as an angiogenic promoter [47,48]. Upon treating with FSK, IL-6 was observed to be down regulated in a dose dependent manner, implying that FSK potentially down regulates IL-6 in cervical cancers. The pro-inflammatory mediators directly correlate with inducible nitric oxide synthase (NOS2), which is an emerging biomarker of aggressive tumours that predicts poor survival in patients with elevated tumour NOS2 expression [49,50,51]. Other clinical studies warrant an improved mechanistic understanding of intratumoral NOS2 regulation and endogenous NO production, which may be therapeutically beneficial. The FSK has induced the downregulation of NOS2 in a dose dependent manner. Similarly the effect of FSK against the apoptotic proteins Bcl-2, Bax, and Caspase-3, reveals its potentiality as the therapeutic modulator, FSK was shown to positively regulate the expression of caspase-3 and Bax were in a dose dependent way and negatively regulate the expression of its counterpart anti-apoptotic protein Bcl2. Full blots are represented in Supplementary Figure S4.

Considering the above findings, it can be deduced that FSK can act as a potential drug to treat various cancers which imparts its effect by deregulating the apoptotic proteins and by transforming the tumour micro-environment, further it serves as a scaffold in designing and developing new drugs.

## Figures and Tables

**Table 1:** Decoy set values computed for pharm 1 and pharm 2.

Parameters	Pharm 1	Pharm 2
Total number of molecules in database (D)	1500	1500
Total number of actives in database (A)	20	20
Total number of hit molecules from the database (Ht)	14	16
Total number of active molecules in hit list (Ha)	13	13
% Yield of active (Ha/Ht)	92	81
% Ratio of actives ((Ha/A) × 100)	65	65
Enrichment Factor (EF)	69.64	60.93
False negatives (A-Ha)	7	7
False positives (Ht–Ha)	1	3
Goodness of fit score (GF)	0.73	0.75

**Table 2:** Comprehensive intermolecular interactions.

Docking Results	Hydrogen Bond Interactions	van der Waals Interactions	π-π/π-alkyl Interactions
Before MD	Leu477: O-H53 (2.9)	Arg115, Phe221, Ile305, Asp309, Thr310, Val369, His480, Ser478	Ile133, Phe134, Trp224, Ala306, Val370, Leu372, Val373, Met374,
After MD	Arg115: HE-O6 (2.0) Ser478:HG1-O7 (1.9)	Phe221, Gln225, Ala307, Thr310, Leu372, Val373, Ala438	Ile133, Phe134, Tpr224, Leu228, Ala306, Val370, Met374, Leu477
Reference	Arg115:HH11-O1 (2.5) Met374:HN-O1 (1.9)	Phe134, Ile305, Ala306, Asp309, Thr310, Leu477, Ser478	Ile133, Phe221, Val370, Val373

## Supplementary Materials

The following are available online at http://www.mdpi.com/2077-0383/7/12/551/s1, Figure S1: Molecular dynamics simulations results, Figure S2: Accommodation of the ligand at the active site of the protein and is observed to be seated at the substrate binding site, Figure S3: The identified compound demonstrates the pharmacophore features of both the models, Figure S4: Blots 1, 2, and 3 were done in parallel with three different SDS-PAGE gels by using same cell lysates. Blots were cropped depending upon molecular size of protein of study. In Blot-3, A was probed with Bcl2 (SC-7380) to see its expression then the blot was treated with harsh stripping process and re-probed with SC-7480 for Bax analysis in blot B; Table S1: 2D structures and molecular dock scores of top nine compounds.
