# Supplementary material for: Natural Compound Modulates the Cervical Cancer Microenvironment—A Pharmacophore Guided Molecular Modelling Approaches
Source: J Clin Med. 2018 Dec 15;7(12):551. doi: 10.3390/jcm7120551 (PMC6306730; doi:10.3390/jcm7120551)
Supplement: Supplementary file 1 [file jcm-07-00551-s001.pdf]

Natural Compound Modulates the Cervical Cancer Microenvironment-A Pharmacophore Guided Molecular Modelling Approaches

Shailima Rampogu[1†], Doneti Ravinder[2†], Smita C Pawar[2*], Keun Woo Lee[1*].

[1]Division of Life Science, Division of Applied Life Science (BK21 Plus), Plant Molecular Biology and Biotechnology Research Center (PMBBRC), Research Institute of Natural Science (RINS), Gyeongsang National University (GNU), 501 Jinju-daero, Jinju 52828, Republic of Korea
[2]Department of Genetics, University College of Science, Osmania University, Hyderabad 500 007, Telangana, India.

† Equal contribution

*Corresponding authors

Supplementary Figure 1

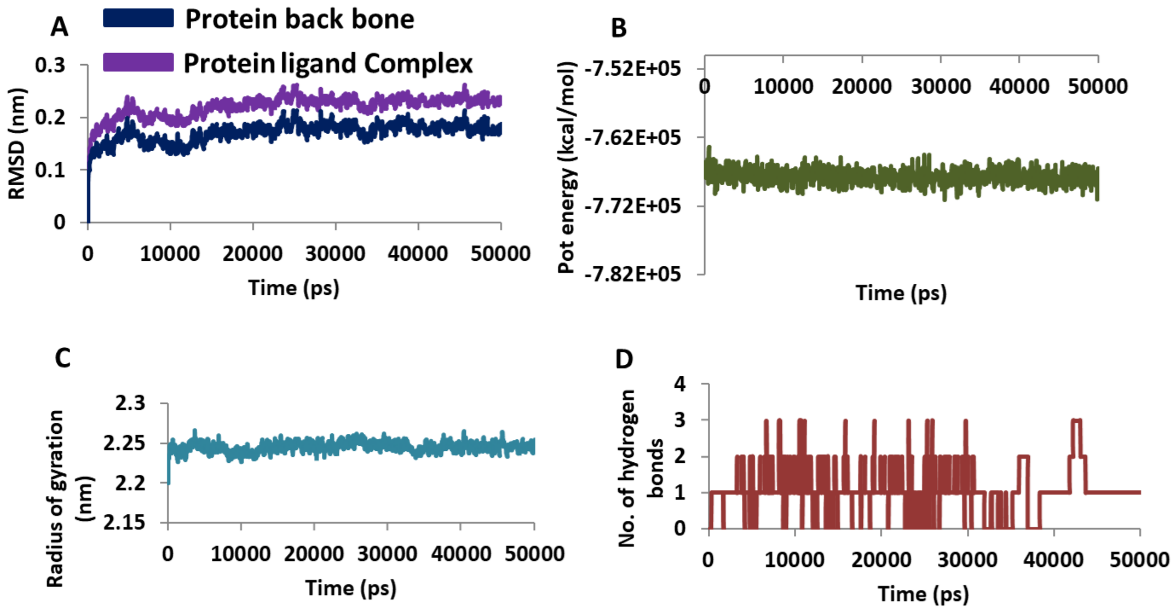

Supplementary Figure 2

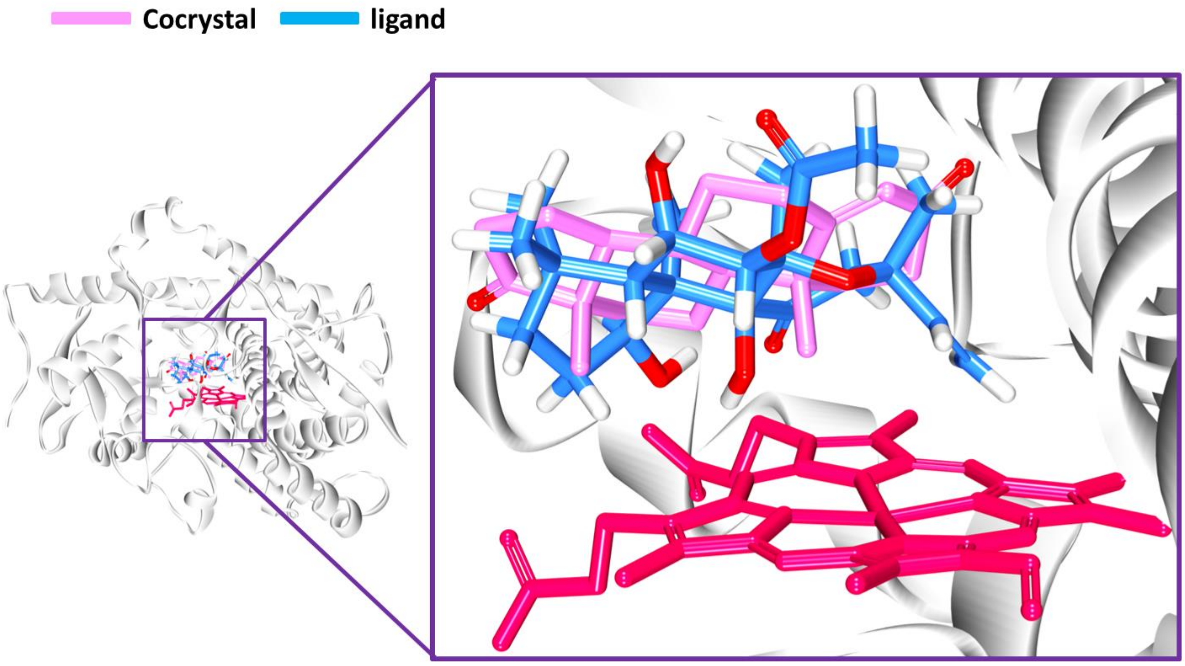

Supplementary Figure 3

FSK_pharm 1 **FSK_pharm 2**

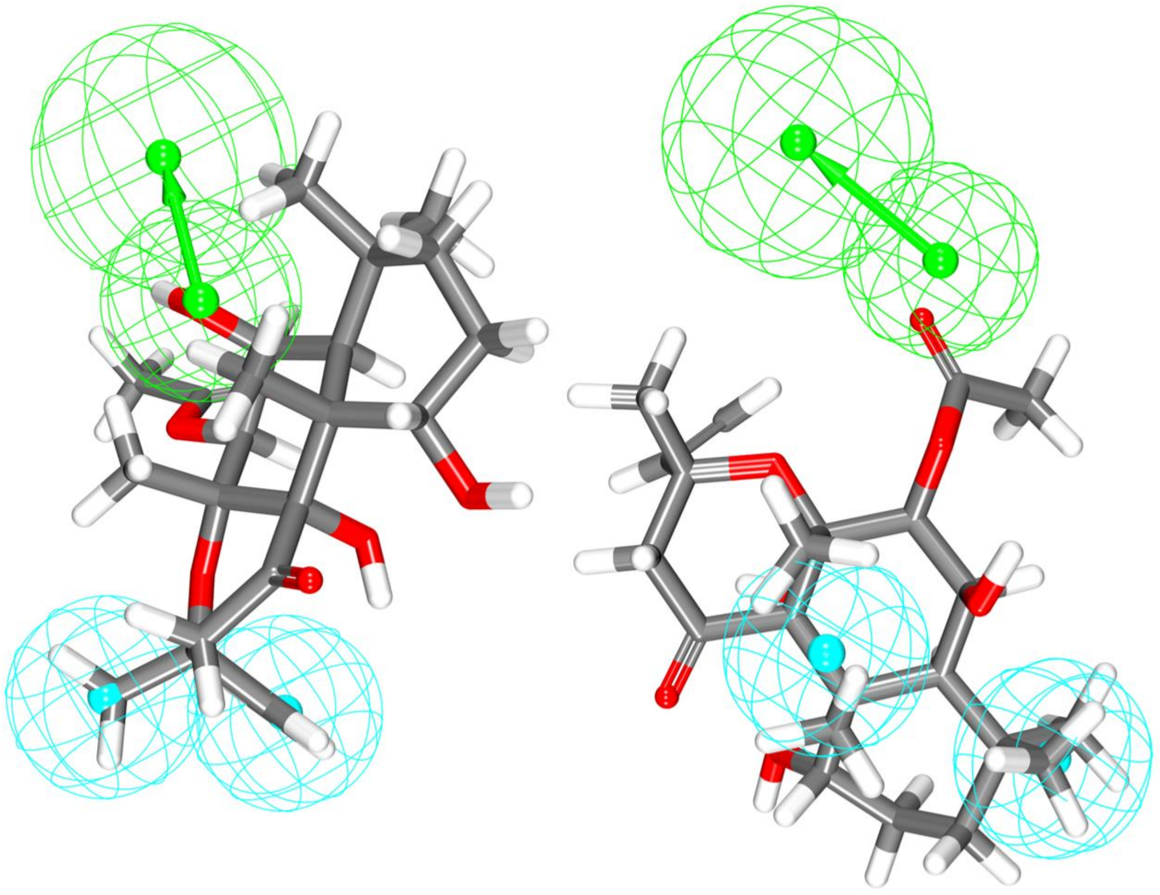

Supplementary Figure 4

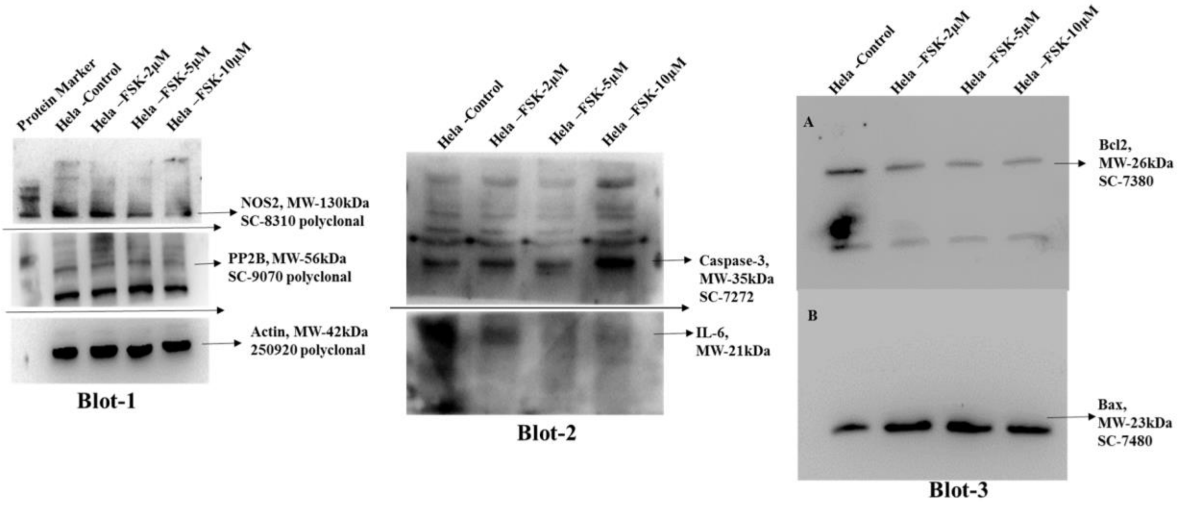

Supplementary Table 1

Compound	Compound structure	-Cdocker interaction energy
1	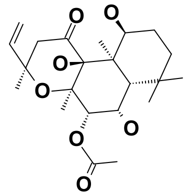	36.10
2	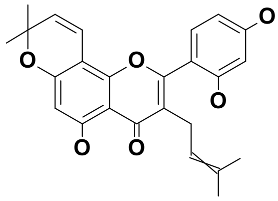	30.22
3	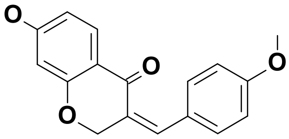	35.66
4	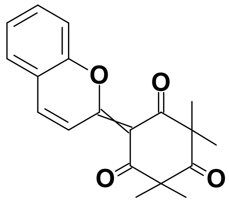	34.77
5	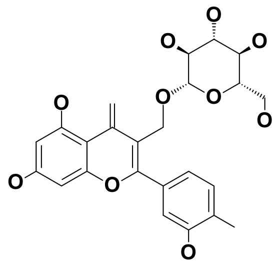	34.74
6	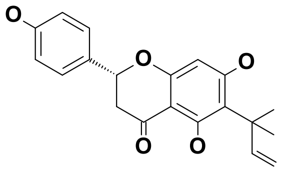	33.89
7	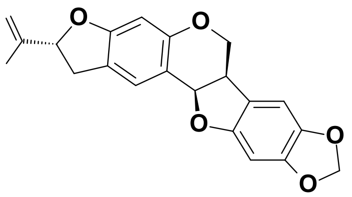	34.05
8	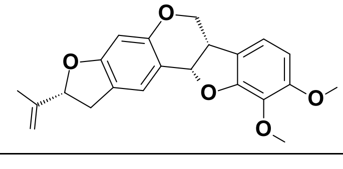	32.56

9	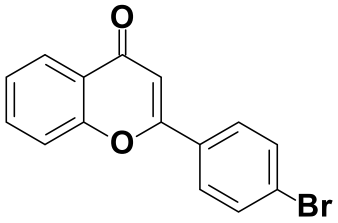	31.96
Reference	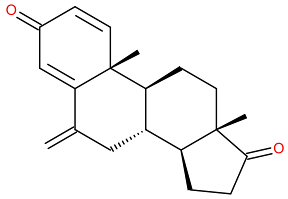	29.97
